# Analysis of Expressed Sequence Tags and Characterization of a Novel Gene, *Slmg7*, in the Midgut of the Common Cutworm, *Spodoptera litura*

**DOI:** 10.1371/journal.pone.0033621

**Published:** 2012-03-28

**Authors:** Wen-yin He, Zhong-chen Rao, Dao-hua Zhou, Si-chun Zheng, Wei-hua Xu, Qi-li Feng

**Affiliations:** 1 Guangdong Provincial Key Lab of Biotechnology for Plant Development, School of Life Sciences, South China Normal University, Guangzhou, China; 2 State Key Laboratory of Biocontrol, School of Life Sciences, Sun-Yat Sen University, Guangzhou, China; Ghent University, Belgium

## Abstract

**Background:**

Out of total 3,081 assembled expressed sequence tags (ESTs) sequences representing 6,815 high-quality ESTs identified in three cDNA libraries constructed with RNA isolated from the midgut of *Spodoptera litura*, 1,039 ESTs showed significant hits and 1,107 ESTs did not show significant hits in BLAST searches. It is of interest to clarify whether or not these ESTs that did not show hits function in *S. Litura*.

**Results:**

Twenty “no-hit” ESTs containing at least one putative open reading frame were selected for further expression analysis. The results from northern blot analysis showed that six of the selected ESTs are expressed in the larval midgut of this insect at different levels, suggesting that these ESTs represent true mRNA products, whereas the other 14 ESTs could not be detected. Homologues of the four larval midgut-predominant genes (*Slmg2*, *Slmg7*, *Slmg9* and *Slmg17*) were detected in the genomes of other lepidopteran insects but not in *Drosophila melanogaster*. A novel gene, *Slmg7*, is expressed at a high level specifically in the midgut during each of the larval stages. *Slmg7* is a single copy gene and encodes a 143-amino acids protein. The SLMG7 protein was localized to the cytoplasm of *Spli*-221 cells.

**Conclusions:**

Six ESTs from the no hit list are transcribed into mRNA and are mainly expressed in the midgut of *S. litura*. *Slmg7* is a novel gene that is localized to the cytoplasm.

## Introduction

Expressed sequence tag (EST) analysis has been widely applied to the studies of gene cloning and expression analysis because of its high reliability. EST analysis of midguts has been reported in several insects, such as *Bombyx mori* [1], *Drosophila melanogaster* [2], *Manduca sexta* [3], *Chironomus tentans* [4], *Mamestra configurata* [5], *Spodoptera frugiperda* [6], *Epiphyas postvittana* [7], *Choristoneura fumiferana* [8], *Anopheles stephensi* [9] and *Plutella xylostella* [10]. However, it is found that there are always certain percentages (ranged from 13% to 30%) of ESTs that show low similarities or no similarity to sequences deposited in the public databases. There are several possibilities for these unidentified ESTs. While sequencing or sequence assembling errors may result in false ESTs, some unidentified ESTs may represent ESTs of real genes that have not been identified or uncharacterized or have not been deposited in the public databases [11]–[12]. For example, they may be real ESTs but are species-specific [13]–[14]; they, therefore, can not be annotated by homolog blasting in other species that have already been sequenced and deposited in the databases.

*Spodoptera litura* is one of the most damaging phytophagous pest insects in tropical and subtropical areas and feeds on a wide range of plants in families of cruciferae, solanaceae, cucurbitaceae and leguminosae. Midgut is a vital organ that is involved in food digestion, nutrient absorption, and immune defense. However, little is known about gene expression in the midgut of *S. litura*. To study EST gene expression patterns of the *S. litura* midgut during metamorphosis, three cDNA libraries were constructed with mRNA isolated from the midguts of the 5^th^ to 6^th^ instar molting larvae (day 0 in 6^th^ instar, L6D0), 6^th^ instar feeding larvae (day 3 in 6^th^ instar, L6D3) and prepupae (day 6 in 6^th^ instar, L6D6) and 6,827 high-quality ESTs have been generated (Qili Feng et al., unpublished). Blast searches showed no hits in the NCBI non-redundant (nr) protein (at E-value<10^−5^) or the nucleotide (nt) database (at E-value<10^−10^) for 1,107 ESTs.

In this study, twenty of these unannotated ESTs containing an open reading frame were randomly selected for further analysis in an attempt to examine if they are expressed in this insect. A novel gene *Slmg7* that encodes a novel protein was then further investigated for its expression and possible physiological function in *S. litura*. The results suggest that some of the ESTs identified in the *S. litura* midguts are Lepidoptera-specific.

## Materials and Methods

### Rearing and sampling of larvae

Second instar larvae of *Spodoptera litura* were provided by the Insect Rearing Facility of the Entomology Institute at Sun Yat-Sen University, Guangzhou, China, and reared on artificial diet until adults in the laboratory conditions (25°C, 60–70% humidity, 12 h light and 12 h dark). The larvae at the selected stages were placed on ice and carefully dissected to isolate different tissues such as midgut, epidermis and fat body. After washing in phosphate buffered saline (PBS), the tissues were stored at −80°C until use for RNA extraction.

Larvae of *Helicoverpa armigera* were obtained from the Entomology Institute at Sun Yat-Sen University; Larvae of *Bombyx mori* Dazao was obtained from The Research and Development Center of the Sericultural Research Institute of the Academy of Agricultural Sciences of Guangdong Province, China. *Drosophila melanogaster* was obtained from Shanghai Institute of Plant Physiology and Ecology, Chinese Academy of Science. The genomic DNA of *Choritoneura fumiferana* was obtained from Great Lakes Forestry Centre, Canadian Forest Service.

### Selection of ESTs and bioinformatics analysis

Total RNAs were extracted using Trizol Reagent (Gibco, USA) and mRNAs were isolated using Oligotex mRNA Kits (Qiagen, USA) from the midguts of the 5^th^ to 6^th^ instar molting larvae (day 0 in 6^th^ instar, L6D0), 6^th^ instar feeding larvae (day 3 in 6^th^ instar, L6D3) and prepupae (day 6 in 6^th^ instar, L6D6). The first strand cDNAs were synthesized through reverse transcription reaction from the mRNAs and used as the templates for double-strand cDNAs synthesis by PCR reaction. T4 DNA polymerase was used to make blunt ends of double-stranded cDNAs and *Eco*R I adaptor was added to the ends of the double-stranded cDNAs, which were then phosphorylated and digested by Xho I. cDNAs of 0.5–1 kb and >1 kb in length were recovered, respectively, using QIAEX II Gel Extraction Kit (Qiagen, USA) and ligated with pBluescript II KS (+) (Stratagene, USA). Competent *Escherichia coli* cells (DH10B) were transformed with the ligation products and the resulting bacteria were plated on the agar medium containing ampicillin. Bacterial clones were randomly selected from the three cDNA libraries and subjected to 5′-single pass sequencing using Applied Biosystems (ABI) 3730 DNA sequencer, generating 6,827 high-quality ESTs (Qili Feng et al., unpublished). DNA sequence data were acquired by Phred (Q = 13) and Phd2fasta. Vector and adaptor sequences were removed using cross_match (-minmatch 15 -minscore 30). The sequences shorter than 100 bp were filtered out. The remaining high-quality sequences were assembled using Phrap under stringent criteria (-minmatch 30 -minscore 45). Consed (Gordon et al., 1998) was used to examine the assembly. Out of the assembled ESTs analyzed 1,107 ESTs did not show hits in the public databases such as nr, nt and dbEST databases of NCBI GenBank (http://www.ncbi.nlm.nih.gov/genbank/), InterPro (http://www.ebi.ac.uk/Tools/pfa/iprscan/) and ExPASy (http://expasy.org/proteomics/protein_sequences_and_identification), twenty assembled ESTs, which ranged from 0.4 to 0.8 kb in length, were selected for the further analysis in this study for their expression by northern blots. Twenty individual longest cDNA clones representing these twenty assembled unannotated ESTs were cloned into pGEM-T vector and sequenced. Putative ORFs of the cDNAs were predicated using ORFfinder program (http://www.ncbi.nlm.nih.gov/gorf/gorf.html).

### RNA extraction and cDNA synthesis

Total RNA was extracted from the whole larvae, midgut, head, Malpighian tubules, hemolymph, epidermis and fat body with Trizol reagent (Takara, Dalian, China) following the manufacturer's protocol. Total RNA was treated with RNase-Free DNase (Takara, Dalian, China) to remove DNA contamination. First strand cDNA was obtained using M-MLV reverse transcriptase (Takara, Dalian, China) and with oligo (dT)18 as primer. First-strand cDNA was generated in a 10 µl reaction solution containing 1.5 µg total RNA, 2× RT buffer, 10 mM dNTP, 20 µM oligo (dT)18, 20 U of RNase inhibitor (Takara, Dalian, China) and 100 U of M-MLV-reverse transcriptase. The reaction was carried out for 90 min at 42°C.

### Southern blot and northern blot analyses

For Southern blot analysis, 10 µg of genomic DNA were digested overnight with different restriction enzymes which did not cut the cloned *Slmg7* cDNA. The resultant DNA fragments were electrophoresed on a 1% agarose gel and transferred to Hybond N1 nylon membrane (Amersham, Piscataway, USA). The membrane was hybridized with a *Slmg7* cDNA probe labeled with α-^32^P dCTP by the Random Primers DNA Labelling Kit (Takara, Kyoto, Japan). Hybridization was performed at 42°C overnight in formamide hybridization buffers. Post-hybridization washes were done in series of low (5×SSPE at 65°C for 15 min, twice), medium [1× standard saline phosphate/EDTA (SSPE), 0.1% SDS at 65°C for 30 min], and high stringency (0.1×SSPE, 0.1% SDS at 65°C for 30 min). The membranes were scanned and photographed in Typhoon TRIO Variable Mode Imager (Typhoon 9400, GE Healthcare Life Sciences, CA, USA).

For northern blot analysis, total RNA was isolated from larval tissues using the standard Trizol reagent (Takara, Dalian, China). Ten microgram of total RNA per sample was separated on 1.0% formaldehyde agarose gels and transferred onto nylon membranes. The probe labeling, pre-hybridization, hybridization and post-hybridization washes were carried out as Southern blot analysis described above.

### Cloning of novel cDNA and rapid amplification of cDNA ends (RACE)

Pairs of degenerate primers (Table 1) were designed on the basis of the original EST sequence to clone the midgut-predominant cDNA directly from mRNA. Total RNA was extracted the midgut of 6^th^ instar larvae and used as template for reverse-transcription PCR (RT-PCR) amplification. Two mg of RNA were treated with 2 units of DNase I to remove trace amounts of genomic DNA and used in each reaction. Reverse transcription was performed using the Reverse Transcriptase M-MLV Kit according to the manufacturer's protocol (Takara, Dalian, China). PCR reaction was performed with 1 cycle of denaturation at 94°C for 1 min, 30 cycles of 94°C for 30 s, 59°C for 30 s and 72°C for 90 s, followed by a 5 min extension at 72°C. PCR products were cloned into pMD-18T (Takara, Dalian, China) and sequenced by Invitrogen Co. (Shanghai, China).

**Table 1:** Primer sequences used for cloning the cDNAs and genes.

Primer ID	Nucleotide sequence
*Slmg2*-F	TAGTCGCTGTATTCTGTT
*Slmg2*-R	AAAAGACGGACTCCCTACG
*Slmg7*-F	CGGCGAAGCTATTAGTGTTG
*Slmg7*-R	TCGTCATCAGCTTGCCAGAG
*Slmg9*-F	GTGATAGCAGACTGATCGA
*Slmg9*-R	GTTGCGGCAGACAATTCTTA
*Slmg17*-F	CAACCCATCACAATGAAATCCT
*Slmg17*-R	TCCAATGGTCCTCAAAAAGTC
*Slmg2*-5R	AGGCGTGGTCTAGCGGTGTCACTGA
*Slmg7*-5R	CTGGCAGCGTGATTTGAGTGACTTGAG
*Slmg9*-5R	TATTCACTGGGGTTCGTATCCGATTCAT
*Slmg17*-5R	ACAACGTGGACGGGGGTAGGCTTGAT

Rapid amplification of cDNA ends (5′ RACE) was accomplished using the BD SMART RACE cDNA Amplification Kit (BD Bioscience Clontech, Mountain View, CA, USA) following the manufacturer's instruction. Gene specific primers for 5′ RACE were designed based on the RT-PCR amplification products or assembled ESTs (Table 1).

### PCR for detection of homologues of *Slmg 2, 7, 9* and *17* in other insects

To detect genomic presence of larval midgut-predominant genes *Slmg2*, *Slmg7*, *Slmg9* and *Slmg17* and their homologues in the genomes of other insects, PCR amplification of these genes was performed using specific primers designed based on the *S. litura* sequences and genomic DNA isolated from *Choritoneura fumiferana*, *Helicoverpa armigera*, *Bombyx mori* and *Drosophila melanogaster*, as well as *S. litura*, respectively. The primers used are listed in Table 1. PCR reaction was performed with 1 cycle of denaturation at 94°C for 1 min, 30 cycles of 94°C for 30 s, 59°C for 30 s and 72°C for 90 s, followed by a 5 min extension at 72°C.

### Quantitative real-time RT-PCR (qPCR) analysis of gene expression

Total RNA was isolated using Trizol Reagent according to the manufacturer's instruction (Takara, Dalian, China) and used to synthesize the first strand of cDNA using the cDNA Synthesis Kits (Takara, Dalian, China). The primers for qPCR were designed based on the sequences of the target genes. qPCR was performed according to SYBRGREEN fluorescent relative quantitative approach [15] using SYBRPremix Ex Tag (Perfect Real Time) (Takara, Dalian, China) and ABI7300 fluorescence quantitative PCR system. Relative expression levels were calculated based on the equation of 2^−ΔΔCT^ [16] according to the manufacturer's instruction. Three independent replicates were conducted for each treatment.

### Production and SDS-PAGE analysis of recombinant protein

Recombinant protein was expressed using an *E. coli* expression system. The 1009-bp *Slmg7* cDNA containing a full-length open reading frame (432 bp) was sub-cloned into a pPROEX HTa expression vector (Life Technologies, Burlington, Canada) between *Eco*R I and *Xho* I restriction sites, with the His-tag at the *N*-terminal end of the target gene. *E. coli* DH5α cells were transformed with the recombinant plasmid DNA pPROEX HTa-*Slmg*7. The His-tagged fusion protein was purified using His-tag resin according the manufacturer's instruction (Novagen, Darmstadt, Germany).

Protein extraction was performed as described by Feng et al. [17] with the following modification. Tissues were homogenized in homogenization buffer (5 ml/g tissue; 50 mM Tris-HCl, pH 7.8, 10 mM EDTA, 15% glycerol, 0.005% phenylthiourea). The homogenate was centrifuged at 10,000 g for 5 min. The supernatant was collected and re-centrifuged again under the same conditions. A mixture of 0.07% β-mercaptoethanol and 10% trichloracetic acid (TCA) in cold acetone were added to the resulting supernatant samples and kept at 4°C for 10 min. The protein extract was then centrifuged at 12,000 g and 4°C for 10 min. The pellets were washed in cold acetone with 0.07% β-mercaptoethanol and centrifuged at 12,000 g and 4°C for 10 min. The resulting pellets were re-suspended in 10 ml lysis buffer consisting of 7 M urea, 2 M thiourea, 4% (w/v) CHAPS, 40 mM Tris, 1% (w/v) dithiothreitol, and centrifuged at 12,000 g and 4°C for 10 min. The supernatant was stored at −80°C until use. The protein samples were denatured by boiling for 5 min after addition of an equivalent volume of 2×SDS loading buffer. SDS-PAGE was performed in 10% polyacrylamide gels. Twenty five microgram protein was used in each of lanes on the gels.

### Cell culture and transfection

To examine the intracellular location of SLMG7, a transfection vector expressing SLMG7/EGFP fused protein was constructed. The Slmg7 cDNA was fused with EGFP in transfection vector pEGFP-1 (Clontech Laboratories Inc., Mountain View, CA, USA) with the GFP fragment at the C-terminal end of the fusion protein. The *Spli*-221 cell line [18] was cultured at 28°C in Grace Insect Medium (Invirogen Co., Guagnzhou, China) containing 10% fetal bovine serum (FBS). *Spli*-221 cells were seeded at 2×10^5^ cells/ml in 2 ml of Grace Insect Medium in 6-well plates. After 12 h incubation, the medium was removed and the cells were washed once with 2 ml of the transfection medium without serum and antibiotics. One milliliter of the transfection medium containing 4 µg transfection vectors and 20 µl lipofectamin (Invitrogen Co., Guangzhou, Guangdong, China) was added to the cell cultures. The cells were incubated in the transfection medium for 8 h and then the transfection medium was replaced with 2 ml of fresh Grace Insect Medium containing 10% FBS and incubated at 28°C. After 24 h in culture, the transfected cells were observed for green fluorescence signals and photographed using a laser confocal microscope.

## Results

### “No-hit” ESTs and their distribution in the three midgut libraries

**Figure 1:**
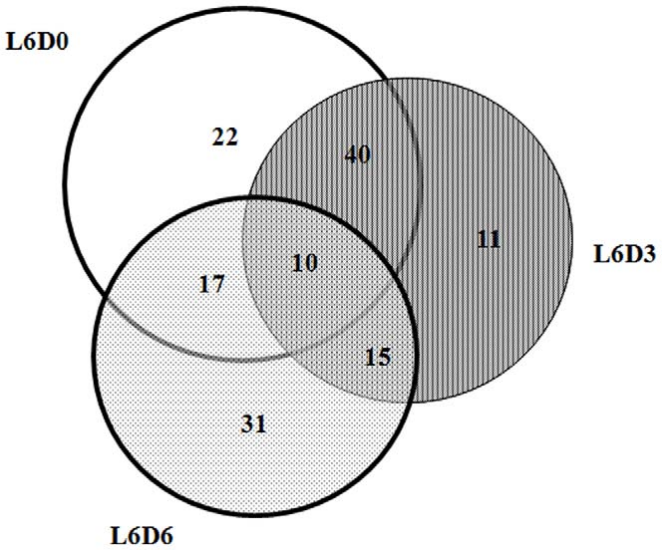
Distribution of “no-hits” assembled ESTs in the three cDNA libraries. L6D0 was a molting phase of 5^th^ instar to 6^th^ instar larvae that did not feed. During this phase, the midgut structure was undergoing remodeling. L6D3 was a larval growth and feeding stage, during which the midgut was actively participating in food digestion and nutrient absorption. L6D6 was a prepupal stage, at which the larvae were not feeding and the midguts were preparing remodeling and transformation from larval to pupal structures.

Totally, 6,815 ESTs were obtained from the three cDNA libraries, among which 2,151 ESTs were from L6D0, 2,238 ESTs from L6D3 and 2,426 ESTs from L6D5. These 6,815 ESTs were assembled into 3,081 unigenes, including 779 contigs and 2,302 singletons. Out of 3,081 unigenes, 1,974 were found to have homologues in public databases, whereas 1,107 unigenes, including 146 contigs and 961 singletons representing 1,579 ESTs, did not match with sequences in the public protein and nucleotide databases at E-values of at <10^−5^ and <10^−10^, respectively (Table 2). The number of these “no-hit” ESTs in L6D0, L6D3 and L6D5 libraries were 630, 509 and 440, respectively. Among the 146 unannotated contigs representing 618 ESTs, 10 contigs were found in all of the three libraries (Fig. 1). Forty contigs were shared in L6D0 and L6D3, 15 contigs in L6D3 and L6D6, and 17 contigs in L6D0 and L6D6 (Fig. 1).

**Table 2:** Numbers of assembled *S. litura* midgut ESTs that had no homologues in the public databases.

	L6D0	L6D3	L6D6	Total
ESTs	630	509	440	1579
Contigs*	89 (195)	76 (204)	73 (219)	146(618)
Singletons	435	305	221	961
Clusters	524	381	294	1107

* The numbers in the parenthesis are for the ESTs that assembled the contigs.

### Bioinformatics analysis of 20 selected ESTs

Twenty assembled ESTs (representing 65 sequences) that had a complete ORF were randomly selected (Table 3). Longest cDNA clones for each of the selected assembled ESTs were identified from the cDNA libraries and were completely sequenced from both the directions. Blast was performed again to check if there are homologues for these selected ESTs in the public databases. Putative ORFs of these twenty unannotated ESTs were predicated using ORFfinder program. Fourteen out of the 20 ESTs, including *Slmg1*, *Slmg2*, *Slmg3*, *Slmg7*, *Slmg8*, *Slmg10*, *Slmg11*, *Slmg12*, *Slmg13*, *Slgm14*, *Slgm15*, *Slgm17*, *Slmg18* and *Slmg20* (Table 3), were predicted to contain an ORF encoding a putative peptide of more that 50 amino acids, thereby suggesting that these are novel protein-encoding sequences. The remaining six sequences contained an ORF that encoded a peptide of shorter than 50 amino acids. Thirteen of the selected ESTs, including *Slmg*2, *Slmg*3, *Slmg*6, *Slmg*7, *Slmg*9, *Slmg*10, *Slmg*11, *Slmg*15, *Slmg*16, *Slmg*17, *Slmg*18, *Slmg*19 and *Slmg*20, had a poly A tail and a stop signal sequence (Table 3).

**Table 3:** Analysis of the selected 20 ESTs of *Spodoptera litura*.

No.	Gene ID	EST ID	Reads No.				EST length	Poly A	Stop signal	Putative ORF	
			Total	L6D0	L6D3	L6D6				Max. (bp)	Max. (aa)
1	*Slmg1*	Contig210	2	1	1	0	643	Y	N	192	63
2	*Slmg2*	Contig194	2	2	0	0	949*	Y	Y	255	84
3	*Slmg3*	Contig678	3	3	0	0	718	Y	Y	205	68
4	*Slmg4*	xw6ca_0002_G09	1	1	0	0	612	Y	N	114	37
5	*Slmg5*	xw6cb_0002_A08	1	1	0	0	522	Y	N	117	38
6	*Slmg6*	xw6cb_0004_E03	1	1	0	0	676	Y	Y	102	33
7	*Slmg7*	Contig577	3	1	2	0	551*	Y	Y	432	143
8	*Slmg8*	xw6cb_0005_D02	1	1	0	0	614	Y	N	168	55
9	*Slmg9*	Contig578	1	1	0	0	1098*	Y	Y	111	36
10	*Slmg10*	xw6cb_0001_B02	1	1	0	0	615	Y	Y	178	59
11	*Slmg11*	xw6cb_0003_A01	1	1	0	0	508	Y	Y	180	59
12	*Slmg12*	xw6ca_0003_H07	1	1	0	0	469	Y	N	372	123
13	*Slmg13*	xw6ca_0004_C02	1	1	0	0	572	N	N	195	64
14	*Slmg14*	Contig553	3	1	2	0	635	N	N	551	183
15	*Slmg15*	xw6za_0003_B07	1	0	1	0	692	Y	Y	177	58
16	*Slmg16*	Contig460	2	0	2	0	739	Y	Y	147	48
17	*Slmg17*	Contig941	36	8	25	3	719*	Y	Y	651	216
18	*Slmg18*	Contig253	2	1	1	0	626	Y	Y	258	85
19	*Slmg19*	xw6za_0005_F08	1	0	1	0	655	Y	Y	114	37
20	*Slmg20*	xw6zb_0005_D05	1	0	1	0	578	Y	Y	153	50
	Total		65	26	36	3					

* Full length sequences.

### Expression patterns of the selected ESTs and cloning of full-length cDNAs

**Figure 2:**
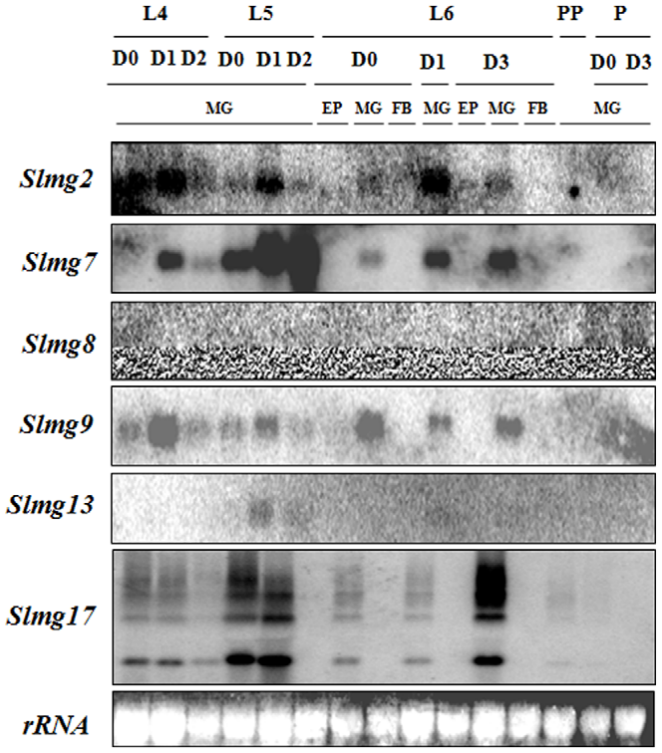
Northern blot analysis of expression of the six selected ESTs from 4^th^ to 6^th^ instar larvae, prepupae and pupae of *S. litura*. Ten micrograms of total RNA isolated from the midgut (MG), epidermis (EP) and fat body (FB) was separated on denaturing 1% MOPS-formaldehyde gel. A ^32^P-labled EST fragment was used as a probe. Ethidium bromide stained rRNA indicates the equal loading of total RNA. L4D0: the day molting to 4^th^ instar; L4D1: day 1 at 4^th^ instar stage; L6D0: the day (white head) of ecdysis from 5^th^ to 6^th^ instar stage; L6D1-L3D3: 1–3 days after ecdysis into 6^th^ instar stage; PP: prepupal stage; P: pupal stage. MG: midgut; EP: epidermis; FB: fat body.

To determine if these 20 selected ESTs were expressed in the insect, cDNAs of these ESTs were labeled with ^32^P-dATP as probes for mRNA expression analysis by northern blots. Northern blots showed that six (*Slmg2, Slmg7, Slmg8, Slmg9, Slmg13 and Slmg17*) out of the 20 ESTs were found to express in larvae or pupae at different stages (Fig. 2). No mRNA expression was detected for the other 14 ESTs (*Slmg1*, *Slmg3*, *Slmg4*, *Slmg5*, *Slmg6*, *Slmg10*, *Slmg11*, *Slmg12*, *Slmg14*, *Slmg15*, *Slmg16*, *Slmg18*, *Slmg19* and *Slmg20*). *Slmg2*, *Slmg7*, *Slmg8*, *Slmg9* and *Slmg13* showed a single mRNA band, while *Slmg17* showed four bands (Fig. 2). *Slmg*8 was detected only in the pupal stage, while *Slmg13* was detected at low levels in 5^th^ and 6^th^ instar larvae. *Slmg2*, *Slmg7*, *Slmg9* and *Slmg17* were detected only in the midgut of the larval stage but not in other tissues (Fig. 2).

**Figure 3:**
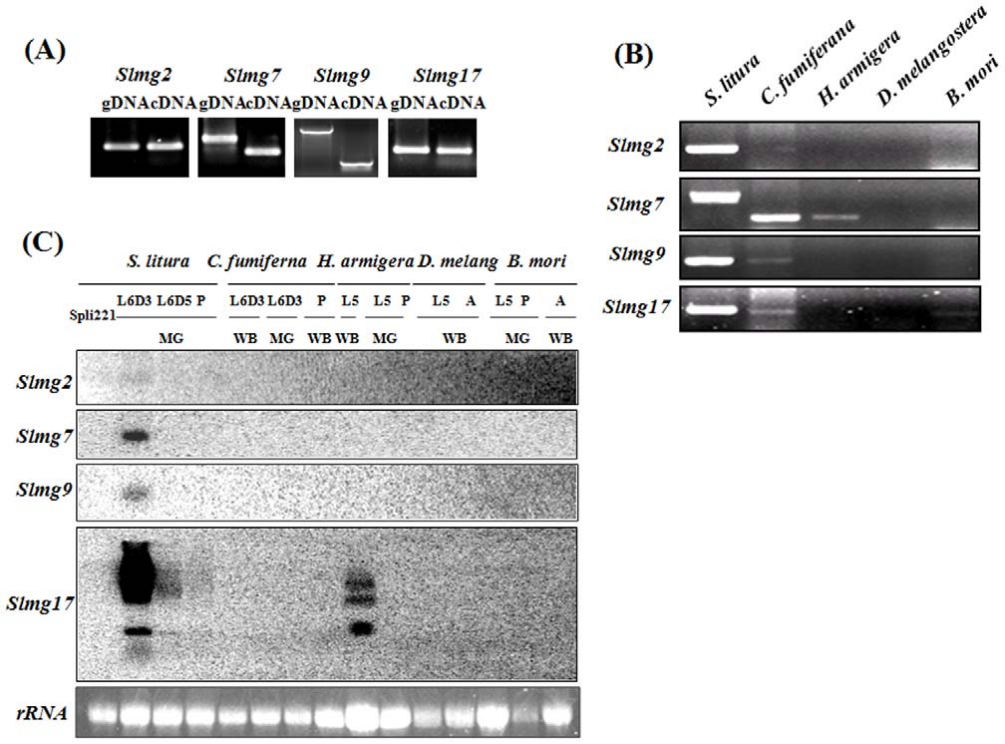
Presence and expression of the four larval midgut-predominant genes. (A) Analysis of presence and expression of the four larval midgut-predominant genes (*Slmg2*, *Slmg7*, *Slmg9* and *Slmg17*) in *Spodoptera litura* by using genomic DNA and cDNA; (B) PCR amplification of the four genes in *S. litura*, *C. fumiferana*, *H. armigera*, *B. mori* and *D. melanogastera* using genomic DNA as templates. (C) Northern blot analysis of expression of the homologous genes in *S. litura*, *C. fumiferana*, *H. armigera*, *B. mori* and *D. melanogastera*. Ten micrograms of total RNA isolated from the midgut (MG) and the whole body (WB) was separated on denaturing 1% MOPS-formaldehyde gel. ^32^P-labeled EST fragments of corresponding *S. litura* genes were used as probes. Ethidium bromide stained rRNA indicates the equal loading of total RNA. *Spli*-221: an embryogenic cell line of *S. litura*; L5: 5^th^ instar larvae; L6D0: the day (white head) of ecdysis from 5^th^ to 6^th^ instar stage; L6D3: 3 day after ecdysis into 6^th^ instar stage; P: pupal stage; A: adult stage.

To further confirm whether or not the four larval midgut-predominant genes (*Slmg2*, *Slmg7*, *Slmg9* and *Slmg17*) were really present and expressed in the insect, RT-PCR amplification of these full-length genes were performed using specific primers designed based on the EST sequences. Genomic DNA and cDNA were used as templates in the PCR amplification. The result shows that the EST-represented genes could be amplified using templates of both genomic DNA and cDNA (Fig. 3A), indicating the genes existed in the *S. litura* genome and expressed as mRNAs in the larval midgut. It is also noted that the sizes of PCR products for *Slmg2* and *Slmg17* were the same for the genomic DNA and cDNA templates (Fig. 3A), indicating that there was no intron in the region of amplicons of these two genes. For *Slmg7* and *Slmg9* the PCR products derived from the genomic DNA templates were larger than that from the cDNA, indicating that there was intron(s) in the *Slmg7* and *Slmg9* genes.

The full-length cDNAs of *Slmg2*, *Slmg7*, *Slmg9* and *Slmg17* cloned above directly from *S. litura* mRNA are summaried in Table 4. The full-length cDNA of *Slmg2* was 949 bp in length and the longest putative ORF was 255 nucleotides. The first 523 nucleotides of the cDNA shared a highest similarity with a DNA sequence, named CL1Contig493 (EZ983023.1), which has not been annotated, in *S. littoralis* at E-value of 10^−63^.

**Table 4:** Open reading frame analysis of the four larval midgut-predominant genes in *Spodoptera litura*.

cDNA ID	Full length (bp)	Longest putative ORF			
		Nucleotide no. (bp)	Amino acid no.	MW (kDa)	pI
*Slmg2*	949	255	84	9.7	9.88
*Slmg7*	551	432	143	15.9	4.84
*Slmg9*	1098	414	137	15.0	8.78
*Slmg17*	719	465	154	16.2	3.49

*Slmg7* full-length cDNA consisted of 551 bp and a polyadenylation signal with a poly(A) tail sequence at the 3′ end. Its longest ORF encoded a 143 amino acid peptide with an estimated molecular mass of 15.9 kDa and a predicted isoelectric point (pI) of 4.84. A putative signal peptide of 18 amino acids was found at its *N*-terminal end. Both nucleotide and protein of this gene show no significant homologues in the database. Because *Slmg7* had a high level of expression specifically in the midgut of the insect, this paper emphasizes characterization of this gene.

*Slmg9* was 1,098 bp in length and its longest predicted ORFs was 414 nucleotides, encoding 137 amino acids. Two parts (around 223–468 nt and 820–960 nt) of this nucleotide sequence show similarity with a serine protease (HM990176.1) in *Mamestra configurata* at E-value of 10^−14^. The putative protein encoded by 82–496 nt of *Slmg9* cDNA was predicted to be an incomplete sequence of serine protease.

The full-length cDNA sequence of *Slmg17* contained an ORF encoding a 154 amino acid protein. Both nucleotide and protein sequences of this gene did not share significant similarity with any sequences in other insect species at E-value of 10^−4^. However, it had a low similarity (E-value equals 10^−3^) to a bacterial mucin (YP502753.1) in *Methanospirillium hungate*.

To examine whether or not these larval midgut-predominant genes (*Slmg2*, *Slmg7*, *Slmg9* and *Slmg17*) are present in the genomes of other insects, PCR amplification of homologues of these genes was performed using specific primers designed based on the *S. litura* sequences and genomic DNA isolated from *Choritoneura fumiferana*, *Helicoverpa armigera*, *Bombyx mori* and *Drosophila melanogaster*. While all these four genes were detected in the *S. litura genome*, homologues of *Slmg2* and *Slmg9* could be amplified from the genomes of *C. fumiferana* and the sizes of PCR products were the same as those in *S. litura* (Fig. 3B). Homologue of *Slmg7* could also be amplified from *C. fumiferana* and *H. armigera* but the sizes of the PCR products were smaller than that in *S. litura*, indicating that no introns probably presented in the genomes of these two species. Homologue of *Slmg17* could be amplified from *C. fumiferana* and *B. mori* and the sizes of the PCR products were the same as that in *S. litura* (Fig. 3B). No homologues of these genes were amplified from *D. melanogaster* suggesting that these novel genes specifically exist in these lepidopteran insects, but the possibility can not be ruled out that the homology of the genes are too low to be amplified by PCR with the primers designed based on the *S. litura* sequences.

Northern blot was performed to examine whether or not these larval midgut-predominant genes expressed in other insects. Total RNA was isolated from *S. litura*, *C. fumiferana*, *H. armigera*, *B. mori* and *D. melanogaster* and tested for the expression of these genes by using *S. litura* cDNA as probes (Fig. 3C). The results indicated that expression of *Slmg2*, *Slmg7* and *Slmg9* were detected only in the midgut of *S. litura* and no expression of these three genes was detected in other species, while *Slmg17* was also detected in the midgut of 5^th^ instar larvae of *H. armigera* (Fig. 3C).

**Figure 4:**
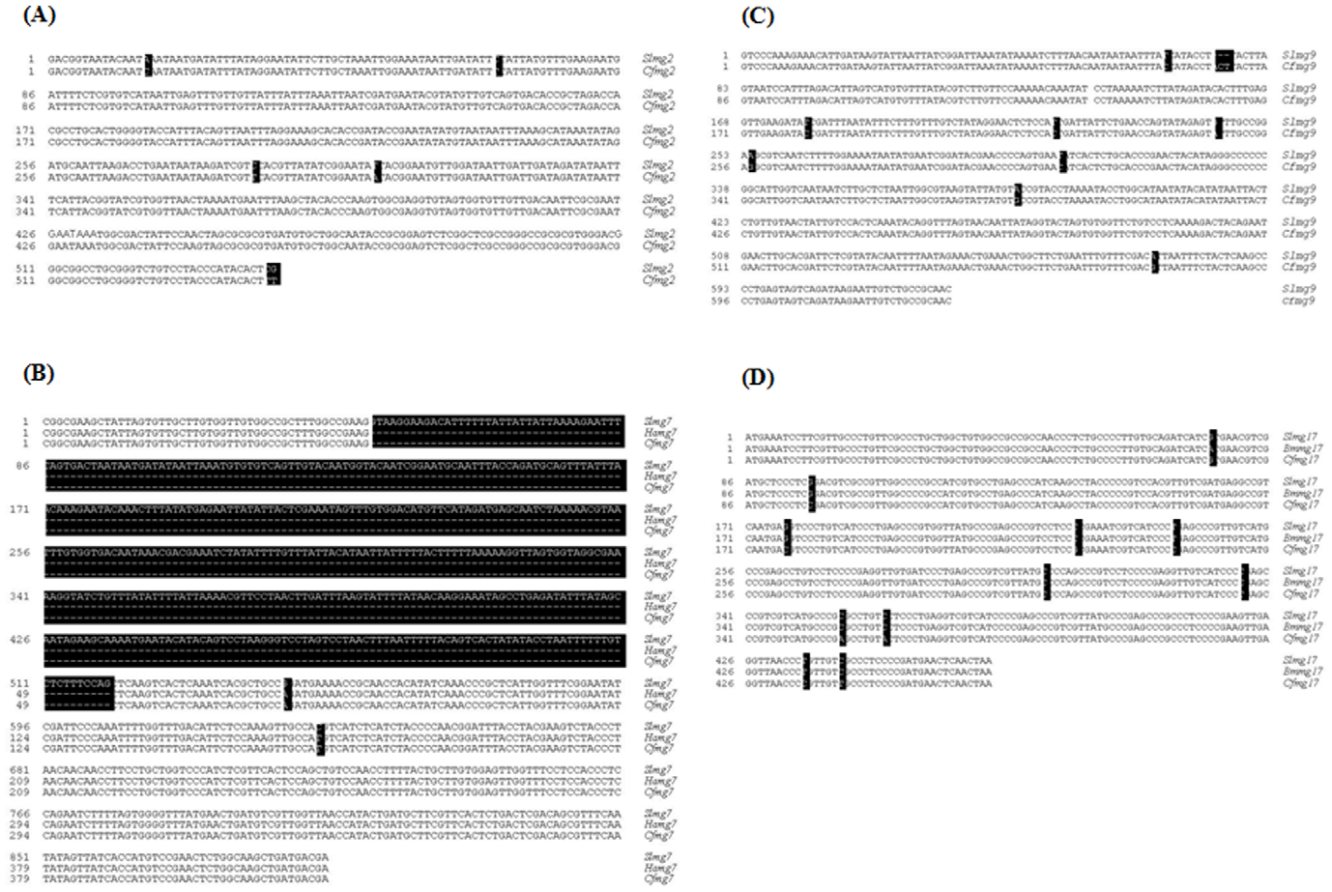
Sequence alignment analysis of the four larval midgut-predominant genes. Alignment of *Slmg2* (A), *Slmg7* (B), *Slmg9* (C) and *Slmg17* (D), with their homologues in other lepidopteran species. Those different nucleotides among the sequences are labelled in black background with white letters. Sl: *Spodoptera litura*; Bm: *Bombyx mori*; Ha: *Helicoverpa armigera*, Cf: *Choristonera fumiferana*. The GenBank accession numbers of these cDNA are JF964956 for *Slmg2*, JF964955 for *Slmg7*, JF964957 for *Slmg9* and JF964954 for *Slmg17*, respectively. The GenBank accession numbers of their homologous genes in other species are JN656431 for *Cfmg2*, JN656432 for *Cfmg7*, JN656433 for *Hamg7*, JN656434 for *Cfmg9*, JN656435 for *Cfmg17* and JN656436 for *Bmmg17*.

Sequence alignment and homologous analyses indicated that *Slmg2*, *Slmg7*, *Slmg9* and *Slmg17* shared high similarities with their homologues in other species in Lepidoptera (Fig. 4), for example, 99.3% between *Slmg2* and *Cfmg2*; 98.3% between *Slmg7* and *Cfmg7*; 98.3% between *Slmg7* and *Hamg7*; 100% between *Hamg7* and *Cfmg7*; 97.8% between *Slmg9* and *Cfmg9*; 99.6% between *Slmg17* and *Bmmg17*; 98.1% between *Slmg17* and *Cfmg17*, and 97.6% between *Bmmg17* and *Cfmg17*.

### Genomic structure of *Slmg7* gene

**Figure 5:**
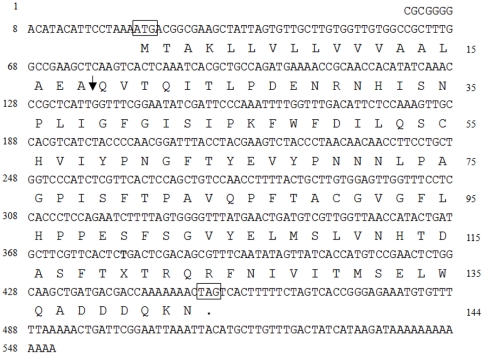
Nucleotide and deduced amino acid sequences of *Slmg7* cDNA. The numbers on the left show the nucleotide sequence and the numbers on the right show the amino acid sequence. The start codon and the stop codon are boxed. The arrow indicates the predicted cleavage site of a signal peptide of 18 amino acids. The GenBank accession number of this cDNA is JF964955.

**Figure 6:**
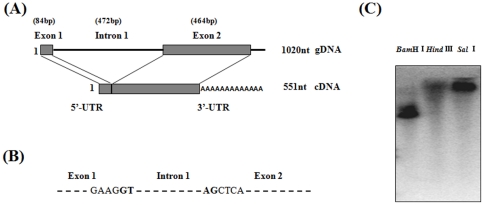
Structure of the *Slmg7* gene. Schematic structure (A), the boundary nucleotides between the exons and introns (B) and Southern blot analysis for copy number (C) of the *S. litura Slmg7* gene. (A) The top panel is for the gene structure and the bottom panel is for the cDNA sequence. The intron sequence is indicated with lines and the exons are indicated with boxes. (B) The intron/exon junction nucleotides are bolded. (C) Ten micrograms of genomic DNA was digested with the restriction enzymes *Bam*H I, *Hind* III and *Sal* I and separated on 1% agarose gel. A ^32^P-labeled *Slmg7* cDNA was used as a probe.

The full-length cDNA *Slmg7* is shown in Figure 5. *Slmg7* gene was amplified by PCR using genomic DNA of *S. litura*. The PCR product of 887 bp in length with a 415 bp fragment within the ORF region was obtained. Sequencing and comparison of the *Slmg7* cDNA and genomic sequences revealed that there were two exons (84 bp and 477 bp) and one intron (472 bp) within the ORF region of the gene (Fig. 6A). The typical intron/exon junction motif (GT-AG) was found in the boundary of the exons and intron (Fig. 6B). To determine how many copies of the *Slmg*7 gene there was in the *S. litura* genome, genomic DNA was digested with the restriction enzymes *Bam*H I, *Hind* III and *Sal* I, which do not internally digest the *Slmg*7 gene, and hybridized with a probe of *Slmg7* cDNA. Southern blot analysis showed that the *Slmg7* cDNA probe detected only a major positive band (Fig. 6C), suggesting that a single copy of *Slmg7* gene is present in the *S. litura* genome.

*In vitro* expression of SLMG7 protein was achieved in a bacterial expression system and the molecular mass of the recombinant SLMG7 protein was estimated as 15.6 kDa (data not shown), closely identical to the predicted size (15.9 kDa) based on the deduced amino acid sequence, indicating that the putative ORF of the *Slmg7* gene could be correctly translated into a full-length protein *in vitro*.

### Developmental expression of the *Slmg7* gene

**Figure 7:**
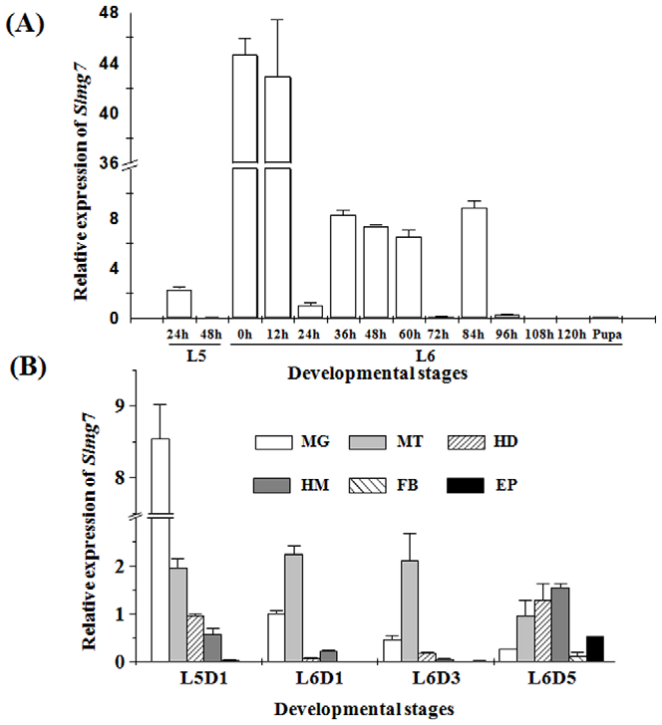
Quantitative real-time RT-PCR (qRT-PCR) analysis of *Slmg7* in different stages and tissues of *S. litura*. (A) Analysis of *Slmg7* expression in the midgut from 5^th^ instar to pupal stages. (B) Analysis of *Slmg7* expression in the midgut, Malpighian tubules, head, hemolymph, fat body and epidermis at 5L1D, 6L1D, 6L3D and 6L5D. The relative expression was calculated by comparing the expression of the *Slmg7* gene over the GADPH (glyceraldehyde-3-phosphate dehydrogenase) gene using the equation of 2^−ΔΔC^_T_ [16]. Values are presented as means ± SD (standard deviation) (n = 9). L5 and L6: 5^th^ and 6^th^ instar larvae, respectively; MG: midgut; MT: Malpighian tubules; HD: head; HM: hemolymph; FB: fat body; EP: epidermis.

To precisely examine developmental expression pattern of the *Slmg7* gene, qPCR analysis was conducted with RNA isolated from the midgut, epidermis, fat body, Malpighian tubules, head and hemolymph of 5^th^ and 6^th^ instar larvae and pupae (Fig. 7). A sharp increase in *Slmg7* mRNA was detected at the first 12 h post ecdysis into 6^th^ instar, followed by a dramatic decline at day 1 of 6^th^ instar stage and then maintained at a stable level during the feeding phases (Fig. 7A). During the larval to pupal transformation and pupal stage, the expression of the *Slmg7* gene became undetectable. The *Slmg7* gene expressed mainly in the midgut, especially in 5^th^ and early 6^th^ instar larvae (Fig. 7B). The *Slmg7* expression was also detected in the Malpighian tubules during the 5^th^ and 6^th^ instar stages. Relatively low or trace expression of the *Slmg7* mRNA was detected in the epidermis, fat body, head and hemolymph in most of the stages tested (Fig. 7B).

### Sub-cellular localization of SLMG7 protein

**Figure 8:**
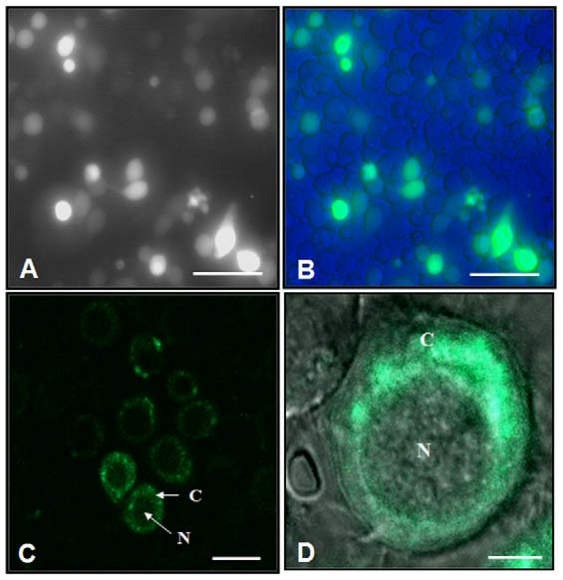
Sub-cellular localization of SLMG7 protein in the transfected *Spli*-221 cells. *Spli*-221 cells were transfected with pEGFP (A and B) and pEGFP/*Slmg*7 (C and D) plasmid DNAs, respectively. The GFP fragment was fused with *Slmg*7 fragment at the *C*-terminal end. The photographs were taken through visible light (A) and fluorescence (B, C and D) filters at 48 h post transfection, respectively. D is an overlapped image under fluorescence and visible light filters. The bars represent 100 µm in (A) and (B), 40 µm in (C) and 10 µm in (D). N: nuclei; C: cytoplasm.

To determine sub-cellular localization of SLMG7 protein, an embryogenic cell line of *S. litura*, *Spli*-221 [18], was transfected with the expression vector pEGFP/*Slmg7*, in which the *Slmg*7 cDNA was fused with EGFP to produce a SLMG7-GFP fusion protein. At 24 h post transfection, more than 60% of the cells showed green fluorescence signals, indicating that most of the cells were effectively transfected by the recombinant plasmid DNA and were expressing the SLMG7-GFP protein (Fig. 8). The confocal fluorescence microscopy observation on the transfected cells revealed that the green fluorescence signal in the control cells, which were transfected with the plasmid pEGFP-1 expressing GFP alone, was seen in both the cytoplasm and nuclei (Fig. 8B). However, in pEGFP/*Slmg7* transfected cells green fluorescence signal was detected mainly in the cytosol and little signal was found in the nuclei (Fig. 8C and 8D).

## Discussion

*S. litura* is one of the most destructive lepidopteran insects in tropical and subtropical areas of the world. Midgut is a digestive organ that plays critical roles in food digestion, nutrient absorption and entomopathogen defense. Significant differences in gene expression patterns during molting, feeding and metamorphosis of the insect reflect functional and physiological alternations in the midgut. It is not surprising to find some novel genes participating in these complicate processes. The EST sequences identified in this study were from the cDNA libraries that represented three consecutive but distinct phases in development of *S. litura*. L6D0 was a phase of larval to larval molting, during which the insects did not feed. During this phase, the midgut structure was undergoing remodeling. L6D3 was a larval growth and feeding stage, during which the midgut was actively participating in food digestion and nutrient absorption. L6D6 was a prepupal stage, at which the larvae stopped feeding and the midgut was preparing structural remodeling and transformation from larval to pupal structures. Out of 3,037 assembled EST clusters, 1,107 (36.5%, representing 1579 ESTs) were found to not be significantly homologous to any sequences in the public databases (Table 1). In a proteomic study of the *S. litura* midgut [19], a total of 2,043 peptides were identified in the midgut of 6^th^ instar feeding larvae (L6D3) by the shotgun ESI-MS analysis and 1,201 (58.8%) peptides could not be annotated. Among these unknown (or unannotated) peptides, 554 (27.1%) had no any homologues in the public protein databases. In this study, the percentages of “no-hit” ESTs in the three cDNA libraries were 29.2%, 22.7% and 18.1%, respectively. It appeared that either at mRNA or protein level, there are always a certain amount of gene products that can not be annotated using the sequence information deposited in the current public databases. This is also found in many large-scale EST projects for different species, including those model organisms whose genomes have been sequenced [1], [6], [8]. There are several possibilities to explain the presence of the unknown or “no-hit” ESTs. First, sequencing error and mis-assembling of ESTs can result in the fault EST sequences, which are actually not encoded by genomic sequences. This problem can be solved by improving the quality of EST sequencing and assembling. Second, it is possible that the cDNA libraries that are used to generate ESTs are poor quality and biased for redundant ESTs. In general, in the non-normalized cDNA libraries, those genes that express at low levels would be less likely to be found by EST sequencing, while abundantly expressed genes are represented and sequenced. This can be overcome by constructing subtracted and unbiased cDNA libraries so that the non-redundant ESTs can be more likely identified. Third possibility is that these “no-hit” ESTs are not protein-encoding sequences and instead they may be regulatory RNA molecules, like micro-RNA [20]. With more and more micro-RNAs are identified, many “no-hit” ESTs may be classified into this group. Fourth possibility is that these ESTs are real protein-encoding genes, but they are species-specific [13] or have low similarities to their homologues in other species that have been deposited in the public databases, therefore they are unique ESTs (genes) for a particular species. After more genomes are sequenced and ESTs are identified in different species, the number of thus “no-hit” ESTs would be reduced.

In the present study, an average 23% of the high quality ESTs of *S. litura* over the three libraries had no homologues in the public databases, including the databases for model insects such as *D. melanogaster*, *A. gambiea*, *Apis mellifera*, *B. mori* and others, whose genomes have been completely sequenced. Because genomic and transcriptomic information of *S. litura* is strictly limited, it is difficult to determine if all of these “no-hit” ESTs are derived from expressing genes. While expression of 14 assembled ESTs out of 20 representing 65 ESTs was not detectible by northern blot analysis, expressions of six ESTs (30%) (*Slmg2*, *Slmg7*, *Slmg8*, *Slmg9*, *Slmg13*, and *Slmg17*) were detected in the larvae (Fig. 2), indicating that these genes did encode and express into mRNAs or regulatory RNA in the insect.

For those ESTs that could not be detected by northern blot analysis, it was possible that they expressed at a too low level to be detected by northern blotting or their expression was stage-specific and the sampling time missed the expressed ESTs. However, it was also likely that they were not expressed at all and were not real protein-encoding sequences. But the expression and the existence of the six “no-hit” genes in the genome in this study clearly suggest that in addition to sequencing and assembling errors, “no-hit” ESTs in an EST project may represent some unannotated species-specific genes, whose homologues have not been found in other species. Based on the results of this study on the four larval midgut-predominant novel genes (*Slmg2*, *Slmg7*, *Slmg9* and *Slmg17*), four possibilities for why these ESTs do not match in high similarity any homologues in the public databases. 1) *Slmg2* were found *in vivo*, but its predicted ORF seems to encode a fragment of 84 amino acids, which is located in the 3′ terminal end of the EST and ended in the poly A tail without a stop codon. Thus, *Slmg2* probably is not a protein-encoding gene *in vivo* and does not translate into a protein, but functions as a small regulatory RNA. 2) *Slmg17* had a low similarity to a bacterial mucin, but not to insect mucins, indicating that *Slmg17* might be an ortholog of the bacterial mucin. However, as a result of evolution, it no longer functions as mucin in insects. 3) *Slmg9* had two fragments at the 5′ and 3′ ends similar to a sequence of serine protease gene of insects. However, there is a 400 bp fragment in the middle between the 5′ and 3′ ends and this sequence insertion may be introduced by genetic recombination and disrupt the ORF for a serine protease, therefore, this gene can not produce a functional protein. 4) *Slmg7* had a complete ORF and can translate into a protein *in vitro*. It is a novel protein-encoding gene specific for *S. litura*, although its biological function remains to be clarified.

Another finding in this study is that expressions of *Slmg2*, *Slmg7*, *Slmg9* and *Slmg17* ESTs appeared to be larval midgut predominant, if not specific, in *S. litura* (Fig. 2), indicating that they may play specific but unknown function(s) in the larval midgut in this species. In addition, homologues of the *Slmg2*, *Slmg7*, *Slmg9* and *Slmg17* genes were amplified from different lepidopteran species by using primers designed based on the *S. litura* sequences, for example, *C. fumiferana* homologue of *Slmg2*, *C. fumiferana* and *H. armigera* homologues of *Slmg7*, *C. fumiferana* homologue of *Slmg9* and *C. fumiferana* and *B. mori* homologues of *Slmg17* (Fig. 3B). These genes appear to be highly conserved and homologous (Fig. 4). However, expression of these homologous genes were not detectable in these species, except *Slmg17* in *H. armigera*, by northern blot analysis when *S. litura* gene probes were used (Fig. 3C), indicating that these homologous genes are not actively expressed during the tested stages in the corresponding insects. This may be one of the reasons for why no homologues of these genes were found in the public databases. The *B. mori* genome has been completely sequenced [21] and *B. mori* homologue of *Slmg17* was amplified when *Slmg17* primers and *B. mori* genomic DNA were used in this study (Fig. 3B), however, homologous sequence was not found in the *B. mori* genome database (http://silkworm.genomics.org.cn/ and http://papilio.ab.a.u-tokyo.ac.jp/genome/).

One question is what functions these novel genes or proteins may play in the midgut of *S. litura*. To explore this question, as the first step, *Slmg7* was first focused in this study. *Slmg7* was a novel gene that specifically expressed in the midgut of *S. litura* larvae (Figs. 2 and 3) and encodes a 143 amino acid protein (Fig. 5). It contains an intron (Fig. 6A) and has a single copy in the genome (Fig. 6C). There was no homologous sequence of *Slmg7* found in the public databases when blast search was conducted at protein level (E-value<10^−5^) or at nucleotide level (E-value<10^−10^). The most possible match was the mouse γ-4-crystallin gene [22], which shared an only 10.6% of similarity at E-value of 10^−3^ with SLMG7 protein. A dramatic increase in the expression level of the *Slmg7* mRNA was detected at the first 12 h after the larvae molted into the 6^th^ instar stage and then declined (Fig. 7B). The protein appeared to locate in cytoplasm of *S. litura Spli*-221 cells (Fig. 8), which is consistent with the fact that a putative signal peptide of 18 amino acids was found at its *N*-terminal end of the protein sequence (Fig. 5). It is still not clear what physiological and molecular function of *Slmg7* may play in the midgut of the insect. Because this gene predominantly expressed in the midgut of larvae, it may play a role in the midgut activity-associated events. RNA interference of *Slmg7* was performed in an attempt of determining its possible function in 6^th^ instar larvae, however, no significant phenotype changes were observed although the levels of *Slmg7* mRNA appeared to be reduced (Data not shown).
